# Clinical and Cost-Effectiveness of PSYCHOnlineTHERAPY: Study Protocol of a Multicenter Blended Outpatient Psychotherapy Cluster Randomized Controlled Trial for Patients With Depressive and Anxiety Disorders

**DOI:** 10.3389/fpsyt.2021.660534

**Published:** 2021-05-14

**Authors:** Harald Baumeister, Natalie Bauereiss, Anna-Carlotta Zarski, Lina Braun, Claudia Buntrock, Christian Hoherz, Abdul Rahman Idrees, Robin Kraft, Pauline Meyer, Tran Bao Dat Nguyen, Rüdiger Pryss, Manfred Reichert, Theresa Sextl, Maria Steinhoff, Lena Stenzel, Lena Steubl, Yannik Terhorst, Ingrid Titzler, David Daniel Ebert

**Affiliations:** ^1^Department of Clinical Psychology and Psychotherapy, Institute of Psychology and Education, Ulm University, Ulm, Germany; ^2^Department of Clinical Psychology and Psychotherapy, Friedrich-Alexander University of Erlangen-Nuremberg, Erlangen, Germany; ^3^Institute of Databases and Information Systems (DBIS), Ulm University, Ulm, Germany; ^4^Medical Informatics, Institute of Clinical Epidemiology and Biometry, University of Würzburg, Würzburg, Germany; ^5^Department of Sport & Health Sciences, Chair for Psychology & Digital Mental Health Care, Technical University Munich, Munich, Germany

**Keywords:** blended therapy, psychotherapy, depression, anxiety, implementation, routine care, E-Mental-Health

## Abstract

**Introduction:** Internet- and mobile-based interventions (IMIs) and their integration into routine psychotherapy (i.e., blended therapy) can offer a means of complementing psychotherapy in a flexible and resource optimized way.

**Objective:** The present study will evaluate the non-inferiority, cost-effectiveness, and safety of two versions of integrated blended psychotherapy for depression and anxiety compared to standard cognitive behavioral therapy (CBT).

**Methods:** A three-armed multicenter cluster-randomized controlled non-inferiority trial will be conducted comparing two implementations of blended psychotherapy (PSYCHOnlineTHERAPY_fix/flex_) compared to CBT. Seventy-five outpatient psychotherapists with a CBT-license will be randomized in a 1:1:1 ratio. Each of them is asked to include 12 patients on average with depressive or anxiety disorders resulting in a total sample size of *N* = 900. All patients receive up to a maximum of 16 psychotherapy sessions, either as routine CBT or alternating with Online self-help sessions (fix: 8/8; flex: 0–16). Assessments will be conducted at patient study inclusion (pre-treatment) and 6, 12, 18, and 24 weeks and 12 months post-inclusion. The primary outcome is depression and anxiety severity at 18 weeks post-inclusion (post-treatment) using the Patient Health Questionnaire Anxiety and Depression Scale. Secondary outcomes are depression and anxiety remission, treatment response, health-related quality of life, patient satisfaction, working alliance, psychotherapy adherence, and patient safety. Additionally, several potential moderators and mediators including patient characteristics and attitudes toward the interventions will be examined, complemented by ecological day-to-day digital behavior variables via passive smartphone sensing as part of an integrated smart-sensing sub-study. Data-analysis will be performed on an intention-to-treat basis with additional per-protocol analyses. In addition, cost-effectiveness and cost-utility analyses will be conducted from a societal and a public health care perspective. Additionally, qualitative interviews on acceptance, feasibility, and optimization potential will be conducted and analyzed.

**Discussion:** PSYCHOnlineTHERAPY will provide evidence on blended psychotherapy in one of the largest ever conducted psychotherapy trials. If shown to be non-inferior and cost-effective, PSYCHOnlineTHERAPY has the potential to innovate psychotherapy in the near future by extending the ways of conducting psychotherapy. The rigorous health care services approach will facilitate a timely implementation of blended psychotherapy into standard care.

**Trial Registration:** The trial is registered in the German Clinical Trials Register (DRKS00023973; date of registration: December 28th 2020).

## Introduction

The effectiveness of psychotherapy in the treatment of mental disorders has been well-documented (1, 2). However, even in countries with a well-developed health care system, treatment rates are low despite the given demand. In Germany, about 28–63% of people with varying mental disorders in need of treatment remain untreated (3). Psychotherapy as one of the first-line treatments for depressive and anxiety disorders (4–6) is provided for only 10–15% of those who receive treatment (7). One reason for this low utilization rate is the shortage of health insurance covered psychotherapies, documented by waiting times of 3–12 months (3). Other reasons might result from conflicting life tasks and challenges to realize time-consuming psychotherapeutic on-site sessions.

Internet- and mobile-based interventions (IMIs) can provide a means of making evidence-based psychotherapeutic interventions available in a timely manner, thereby contributing to reducing the shortage of care (8, 9). The aim is not only to provide information on possible causes, symptoms, and courses of mental disorders, but also to provide parts or the entire psychotherapeutic process digitally (8). The research on IMIs has so far almost exclusively focused on stand-alone IMIs, i.e., online interventions that are used as an alternative to on-site treatment (8, 9). Numerous clinical trials summarized in several meta-analyses have now shown the effectiveness of IMIs in the treatment of mental disorders (8), particularly well-studied in depressive disorders and anxiety disorders (10, 11). Thereby, guided IMIs for mental and somatic disorders are seemingly as effective as the respective on-site treatments (12). However, the evidence refers to participants who are willing to be treated via IMIs. Studies on the acceptance of IMIs show that this only applies to a small part of the target population regarding both patients (13–16) and therapists (17, 18). In addition, the exclusive remote psychotherapeutic treatment of mental disorders, as is the case with stand-alone IMIs, is largely restricted in Germany by the applicable professional regulations.

Blended psychotherapy, i.e., the combination of online intervention elements with standard psychotherapeutic care, is a rather new field of research (19–22). Recent surveys and qualitative studies amongst psychotherapists indicate that blended therapy approaches would be acceptable considering perceived advantages over conventional psychotherapy, including e.g., bridging distances, flexibility, patient empowerment, and therapist support by standardized materials (17, 18, 23, 24). Acceptance rates are seemingly higher amongst psychotherapists with a background in cognitive behavioral therapy (CBT) compared to other therapeutic backgrounds (17, 18).

From a conceptual point of view, blended therapy approaches can be subdivided into (1) sequential and (2) integrated blended therapy concepts focusing on (a) maximizing the effectiveness of psychotherapy or (b) maximizing the efficiency of psychotherapy (19). Examples for sequential blended therapy concepts are IMIs provided prior to on-site psychotherapy, e.g., during waiting-time (25), or IMIs following on-site psychotherapy, e.g., as inpatient aftercare and relapse prevention (26–28).

Regarding integrated blended therapy, Berger et al. (29) examined whether an Internet-based self-help intervention, when used adjunctive to standard psychotherapy, has an additional effect compared to standard psychotherapy for depression. In this randomized controlled trial, integrated blended therapy was superior over standard psychotherapy (*d* = 0.51; *n* = 98). Similarly, Zwerenz et al. (30) documented an incremental effectiveness (*d* = 0.44; *n* = 229) of an Internet-based self-help program in addition to psychodynamic inpatient psychotherapy for depression compared to inpatient psychotherapy only.

IMIs could also be used to optimize the efficiency of psychotherapy. It has been well-established that therapeutic guidance is an active component of IMIs, however, with a yet to be examined ceiling effect from which onward more therapist time does likely not translate into clinically significant higher therapeutic benefits for the average patient (31, 32). Hence, one possible way of implementing blended therapy is to iteratively provide standard psychotherapy combined with Internet- and mobile based self-help modules, with the assumption of non-inferiority. As such, blended therapy could represent a means of providing psychotherapy to more patients in need, against the background of restricted resources as present in most health care systems around the world.

PSYCHOnlineTHERAPY aims to examine the potential of integrated blended therapy by comparing standard CBT-focused outpatient psychotherapy (CBT_standard_) with two implementation variants of integrated blended therapy, (1) PSYCHOnlineTHERAPY_fix_ as a standardized blended therapy concept combining equal numbers of standard therapy sessions and online intervention modules and (2) PSYCHOnlineTHERAPY_flex_, providing therapists with the means of combining standard therapy with online interventions modules as perceived fitting for the respective therapy process at hand. In more detail, the project aims to examine:

1. the non-inferiority of PSYCHOnlineTHERAPY_fix/flex_ in comparison to CBT_standard_.
2. the cost-effectiveness of PSYCHOnlineTHERAPY_fix/flex_ in comparison to CBT_standard_.
3. the safety of PSYCHOnlineTHERAPY_fix/flex_ in comparison to CBT_standard_.
4. qualitative and quantitative details of the implementation variants with regard to acceptance, feasibility, barriers, and facilitators to identify optimization potential.
5. moderators and mediators of the therapy success as well as potential risks and side effects.

## Methods

### Study Design

A three-armed multicenter large-scale pragmatic, cluster-randomized controlled trial (cRCT) will be conducted comparing the clinical and cost-effectiveness of two implementations of blended therapy (PSYCHOnlineTHERAPY_fix/flex_) compared to standard CBT (CBT_standard_). Quantitative trial outcomes will be complemented by qualitative interview data on the acceptance, feasibility, and optimization potential of PSYCHOnlineTHERAPY in order to gain in-depth insights in participants' experiences. A smart-sensing sub-study, examining ecological day-to-day digital behavior variables via passive smartphone sensing, aims to provide psychotherapy process insights (33).

This clinical trial has been approved by the ethics committee of the German Psychological Society (DGPs no. BaumeisterHarald2020-07-29VADM) and will be reported in accordance with the Consolidated Standards of Reporting Trials (CONSORT) Statement 2010 and the extensions for reporting pragmatic trials, non-inferiority trials, cluster randomized trials, multi-arm parallel group trials, and trials on psychological interventions (34–39). Qualitative data analyses will be reported following the Consolidated Criteria for Reporting Qualitative Studies (COREQ) checklist (40). Cost-effectiveness analyses will be reported according to Consolidated Health Economic Evaluation Reporting Standards statement [CHEERS; (41)] and the guidelines from the International Society for Pharmacoeconomics and Outcomes Research [ISPOR; (42)]. This trial protocol was created according to SPIRIT guidelines (43). The study has been registered in the German clinical trial register under DRKS00023973.

### Participants and Procedure

#### Cluster Definition

The trial will be conducted in psychotherapy outpatient practices in South-West Germany (Baden-Wuerttemberg) that take part in the PNP (Psychotherapy, Neurology, Psychosomatic, and Psychiatry) selective health care services contract of the health insurance companies AOK Baden-Wuerttemberg and Bosch BKK, managed by MEDIVERBUND AG according to §73c SGB V. This contract defines (amongst others) specific psychotherapy services for patients as outlined below. Clusters are defined by psychotherapy outpatient practices that are run by licensed psychotherapists who are PNP contract partners (i.e., authorized to bill according to the PNP contract; https://www.mediverbund-ag.de/file/4922). Psychotherapists who are not PNP contract partners themselves may participate in the study, if they are employed in a practice owned by a PNP contract partner. More than one therapist per practice is allowed to participate in the study.

Psychotherapists are eligible for inclusion in case of a given informed consent and if they (a) are actively working as a psychological psychotherapist, a medical specialist for psychiatry and psychotherapy or psychosomatic medicine and psychotherapy, another physician working as psychotherapist, or a children and adolescent psychotherapist, (b) are (employed in a practice owned by) a PNP contract partner, (c) hold a CBT license, (d) are available during recruitment and assessment period (self-report regarding no already known time-offs), and (e) are capable of including 12 patients into the study during the recruitment period (18 months; self-report). Enrolment and opening of clusters will take place over the entire recruitment period in order to reach the recruitment target of 900 patients. The recruitment target is a median of 12 patients per participating psychotherapist in order to achieve recruitment of 900 patients.

#### Inclusion and Exclusion Criteria of Patients

Psychotherapy outpatients of enrolled psychotherapists are eligible for inclusion in case of a given informed consent and if they (a) are ≥ 18 years, (b) have a depressive disorder or anxiety disorder diagnosis eligible to be treated under the PNP contract (medical record of an ICD-10 F32/33.1-0.3; F34.1; F40.00/0.01; F40.1; F40.2; F41.0-0.3), (c) have a health insurance contract with AOK Baden-Wuerttemberg or Bosch BKK as part of the PNP contract according to §73c SGB V, (d) complete the baseline assessment (online assessment and telephone-based standardized clinical interview), (e) have Internet-access and an Internet-capable device (self-report), (f) have sufficient knowledge of the German language (therapist rating), (g) have no ICD-10-F2 diagnosis (therapist rating) as IMIs are not well-examined for this patient group yet, and (h) show no clinical reasons for exclusion (psychotherapist rating). Exclusion criteria are kept at a minimum in this effectiveness trial embedded in standard psychotherapeutic outpatient care. Suicidal tendencies are not defined as exclusion criteria and will be therapeutically handled by the treating psychotherapist according to established standards for crises interventions in standard psychotherapeutic care. In case of acute suicidal tendencies psychotherapists might judge patients as not clinically suitable for blended therapy (criterion h). Respective therapist decisions will be recorded weekly.

### Recruitment

Initial recruitment of psychotherapists started in July 2020 and is expected to be finished in July 2021, supported by MEDIVERBUND AG as well as the collaborating professional associations MEDI Baden-Wuerttemberg e.V., Freie Liste der Psychotherapeuten and Deutsche Psychotherapeutenvereinigung (DPtV). First patient in is expected to be included in January 2021. Psychotherapists taking part in the PNP contract were contacted via e-mail and invited to one of four online information events. Furthermore, psychotherapists can express their study interest at www.psychonlinetherapie.de. After informed consent has been given and inclusion and exclusion criteria confirmed, eligible psychotherapists complete baseline assessment. Psychotherapy outpatient practices are consecutively randomized to one of the three trial arms. Psychotherapists are subsequently invited to a 1-day training, which will be tailored to the respective trial arm (PSYCHOnlineTHERAPY_fix/flex_; CBT_standard_). MEDIVERBUND AG assigns a practice structural feature for billing project-specific blended therapy services in accordance with the PNP contract.

**Figure 1:**
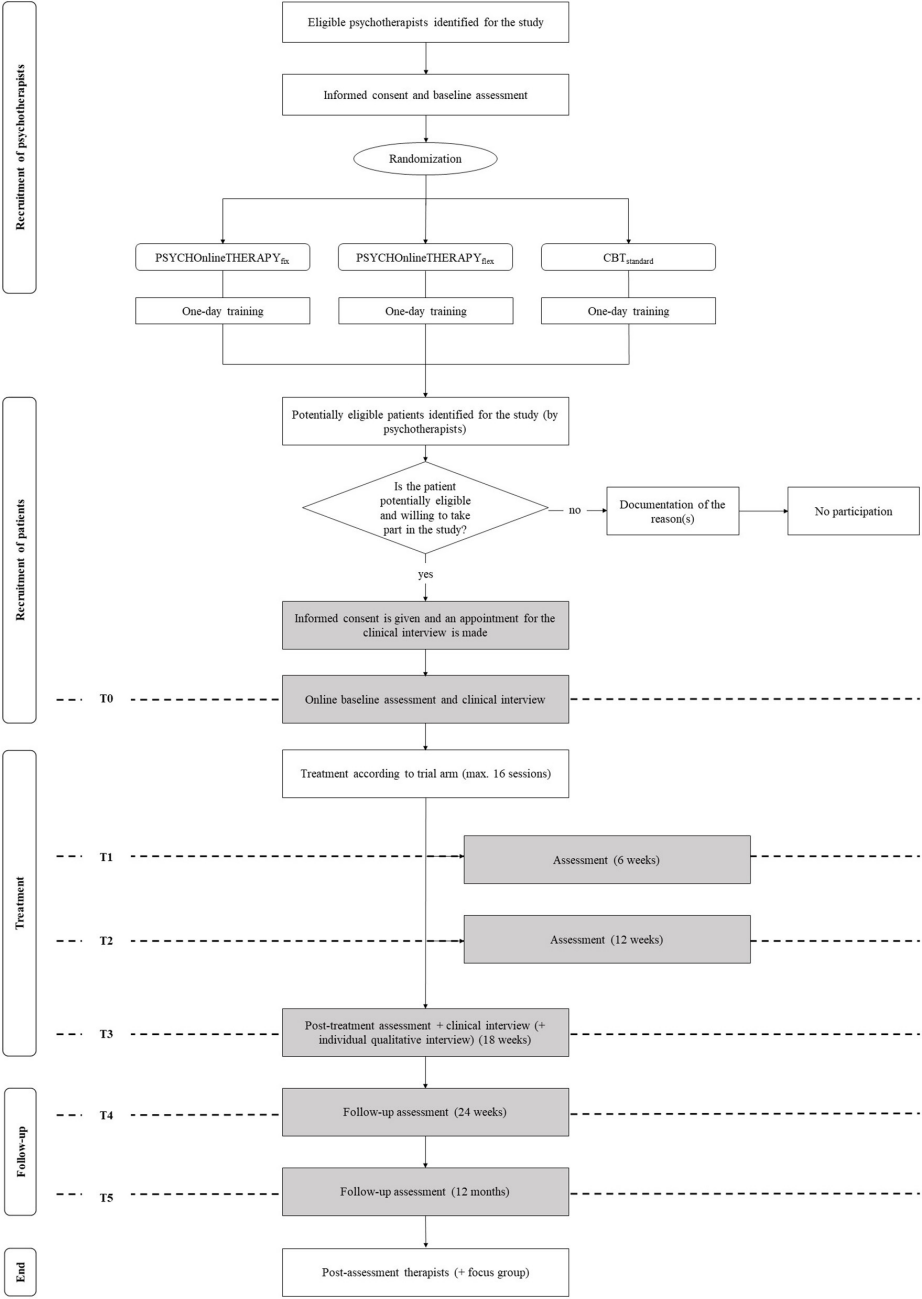
Flow chart.

Once psychotherapists are allocated to one of the three trial arms and trained for intervention and study protocol adherence, patient recruitment starts. This is expected to take place from January 2021 until June 2022. The psychotherapists assess if patients are potentially eligible for the trial. If this is the case, patients are informed by their psychotherapists about the possibility to take part in PSYCHOnlineTHERAPY. Interested patients will give their informed consent and schedule an appointment for the telephone-based clinical interview online on a tablet provided for study purposes only in the psychotherapy practice. Patients are then invited for the online baseline assessment and the baseline clinical interview is conducted. Once the baseline assessments have been completed, patients enter the trial and are treated for their condition according to trial arm allocation of their psychotherapist following the intervention rational as described below. Follow up assessments will take place as outlined in the flow chart (Figure 1).

### Randomization, Allocation, and Masking

Psychotherapist outpatient practices will be consecutively randomly allocated to (a) PSYCHOnlineTHERAPY_fix_, (b) PSYCHOnlineTHERAPY_flex_, or (c) CBT_standard_ and informed about group membership via e-mail. Randomization will take place at a psychotherapist practices level. That means, therapists in joint practices are randomized jointly into one of the three trial arms to avoid trial arm contamination. Randomization will be conducted by an independent researcher who is blind regarding the study conditions. Whereas, blinding of psychotherapists is not possible, data collectors are blinded regarding treatment condition. Treatment condition is only known by the study personnel administering allocated treatments to psychotherapy practices. Randomization will be stratified by the number of therapists per practice (single therapist vs. more than one therapist). Two randomization lists will be generated by using the web-based programme Sealed Envelope (www.sealedenvelope.com). Randomization will happen on an individual level and an allocation ratio of 1:1:1 will be performed. In case of dropouts and if it becomes apparent that more than 75 therapists are needed to reach the sample size of *N* = 900 patients, additional therapists will be randomized.

### Intervention

PSYCHOnlineTHERAPY provides the mean of combining standard psychotherapeutic care as described below (see control condition) with Internet- and mobile-based intervention modules. Based on psychotherapy practice study arm allocation patients receive either PSYCHOnlineTHERAPY_fix/flex_ or CBT_standard_.

All patients with a diagnosed depressive or anxiety disorder as defined in the inclusion criteria can take part in a psychotherapy reimbursed based on the PNP contract. In adult psychotherapy, acute care of up to 10 h is reimbursed (as of October 20th 2020) with 120 €. Initial treatment of a maximum of 20 h is reimbursed at 115 €. Further treatment is possible with up to 30 sessions (105 €). The first max. 16 psychotherapy sessions are defined as trial intervention of PSYCHOnlineTHERAPY. Thereby, the trial definition of 16 sessions follows both trial feasibility considerations as well as psychotherapy dose-response findings, with 4–24 sessions being reported as optimal dose in routine treatment settings (44). Patients of all three trial arms will receive standard PNP-based psychotherapy following the first 16 sessions in case of still existing need of psychotherapy as defined by the therapist. As only psychotherapists with a CBT background are eligible for this trial, PNP-based psychotherapy will be CBT-based.

#### Control Group—CBT_standard_

Patients enrolled in PSYCHOnlineTHERAPY and allocated to the control group will receive standard psychotherapy as described before, following the obligatory diagnostic process in the first few sessions (Table 1). Thereby, therapy follows standard care without a predefined treatment protocol. Details of the psychotherapy provided will be assessed via therapy documentation sheets in order to provide a *post-hoc* description of standard psychotherapy as provided in standard care.

**Table 1:** PSYCHOnlineTHERAPYfix/flex vs. CBTstandard.

**PSYCHOnlineTHERAPY_**fix**_**	**PSYCHOnlineTHERAPY_**flex**_**	**CBT_**standard**_**
on-site diagnostics, indication for psychotherapy and evaluation of suitability for PSYCHOnlineTHERAPY as well as informed consent		
max. 16 sessions in a fixed order alternating an online-intervention module followed by a standard psychotherapy session	max. 16 online or standard sessions, amount and order of online and standard sessions as defined by their therapists	max. 16 standard psychotherapy sessions
end of PSYCHOnlineTHERAPY (standard PNP-based psychotherapy following the first 16 sessions in case of still existing need of psychotherapy as defined by the therapist)		

#### Intervention Groups – PSYCHOnlineTHERAPY

Patients allocated to the intervention group will receive PNP-based psychotherapy as aforementioned, combined with Internet- and mobile-based modules (Table 1).

PSYCHOnlineTHERAPY was developed by the Department of Clinical Psychology and Psychotherapy, Ulm University. The content has been specifically tailored to the needs of psychotherapy outpatients as well as their psychotherapists based on experience from a multitude of prior Internet- and mobile-based intervention development processes and clinical trials [e.g., (29, 45–47)]. Patient and psychotherapist feedback on former versions of the intervention modules has been used to further optimize the modules. The overall web-design was revised based on persuasiveness principles (48). Various interactive design elements such as videos, audio files, pictures, and exercises are included in order to optimize user experience and facilitate intervention adherence.

Intervention content consists of seven Internet- and mobile-based modules for depressive disorders, seven for each of the included anxiety disorders, 22 transdiagnostic modules as well as one introductory and one closing module (Table 2) with an estimated proceeding duration of 45–60 min each. They are based on CBT-principles including psychoeducation, many exercises and homework assignments to promote transfer into everyday life. To illustrate therapeutic principles and exercises within the intervention, fictional patients are introduced in the beginning of the intervention and used to illustrate processes, challenges, and possible solutions throughout the intervention modules.

**Table 2:** Intervention content.

**Modules**	**Content**
Introduction	Platform features, presentation of example patients
**Depression modules**	
Psychoeducation	Introduction, symptoms, Lewinsohn's depression model
Lifeline and therapy goals	Drawing lifeline, risk factors, resources, therapy goal setting
Activities	Depression spiral, activity planning
Depression pitfalls	Behavioral patterns, self-observation, problem solving skills
ABC model	Presentation of the model, individual formulation of components
Cognitive restructuring	Beneficial and impeding thoughts, consequences and connection with emotions, restructuring methods
Emotions	Introduction to and cultural rules of emotions, components of emotion
**Anxiety modules**	
Psychoeducation: development of anxiety^*^	Introduction, symptoms, anxiety levels, diathesis-stress model
Psychoeducation: maintenance of anxiety^*^	Vicious circle, individual formulation of initiating and maintaining factors
Dealing with anxiety reactions	Safety seeking and avoidance behavior, perception control, exercises
Anxiety process^*^	Situation exploration, fear hierarchy, worst possible consequences
Motivation	Cost-benefit relation, neglected activities, goal setting
Confrontation^*^	Confrontation therapy, protocol, exposition exercises in sensu and *in vivo*
Pleasant thoughts	Reflection of thoughts, stress reducing thoughts, exercises
Closing module	Resources, goal setting, emergency plan, motivation
**Transdiagnostic modules**	
Mindfulness	Introduction, effects of mindfulness, exercises
Physical activity	Importance, recommendations, assessment, goal setting, everyday activity
Sleep	Healthy sleep, sleep disorders, stimulus control exercises
Social competence training I	Social situations and competence, confident behavior, asserting rights
Social competence training II	Social situations and competence, confident behavior, recognizing emotions, managing relationships
Grief	Grief reaction, secondary losses, rumination, detrimental thoughts
Pain	Understanding pain, activity despite pain, rumination, relaxation
Relaxation	Introduction, relaxation and meditation exercises
Relationship and sexuality	Communication, needs, relaxation, massages
Self-esteem and self-image	Importance of self-esteem and self-image, elevating self-esteem, values
Self-compassion	Importance of self-care, learning self-compassion
Loneliness	Loneliness vs. being alone, dealing with loneliness, building relationships, exercises
Gratitude	Importance of gratitude, learning gratitude
Perfectionism	Illusion of perfection, origin and consequences, tolerate and accept imperfection
Procrastination	Understanding procrastination, connection with Internet, importance of self-regulation, exercises to overcome procrastination
Substance use	Problematic substance use and reflection, distraction, alternative behavior
Stress	Meaning of work, connection between work-related stress and mental illness, problem- and emotion-focused stress management
Social media	Problematic usage, SORCK-Model, alternative activities, strategies of self-regulation
Somatoform symptoms	Understanding health and illness, connection between stress and physical complaints, ABC model, physical activity
Acceptance	Short-term problem-solving strategies, primary vs. secondary suffering, learning acceptance
Values and goals	Definition of individual values, goal setting, beneficial key assumptions
Stigma	Diagnostic label, self- and public-stigma, sharing diagnosis

* *Individual modules for agoraphobia, generalized anxiety disorder, panic disorder, social phobia, and specific phobia*.

The intervention is available to participants on eSano, an open source e-health platform developed by Ulm University for providing a technological infrastructure to create and deliver a multitude of IMIs. The platform is divided into three sub-platforms. Intervention content is designed and created in the web-based Content Management System. The intervention is then made available to participants in the cross-platform patient application (web-based, Android, iOS). During the intervention therapeutical guidance can be provided using the web-based e-coach platform. Communication and data transfer between all sub-platforms are end-to-end encrypted with TLS. All eSano systems are located in an isolated network environment, whose interfaces to adjacent networks are regulated by firewalls with appropriate rules. These rules are defined to allow a necessary minimum of communication. The platforms are developed oriented on the requirements of the German Medical Devices Act and the Medical Device Regulation (MDR). Thus, the software development and validation process takes into account the IEC 62304 (safety class B), the GAMP5 (category 4), the General Principles of Software Validation of the FDA as well as the Pharmaceutical Inspection Cooperation Scheme (PIC/S) 11-3.

PSYCHOnlineTHERAPY modules can be used by therapists and patients in varying forms, operationalized in the present study in two versions:

PSYCHOnlineTHERAPY_fix_: Patients receive alternating online-intervention modules and standard psychotherapy sessions with a fixed ratio of max. eight online and eight standard sessions. Thereby, therapists are free to choose amongst the available intervention modules and in their decision of module order.

PSYCHOnlineTHERAPY_flex_: Patients receive a flexible number of up to 16 online or standard sessions as defined by their therapist. Thereby, therapists are free to choose amongst the available intervention modules and in their decision of module order as well as frequency.

Both conditions, PSYCHOnlineTHERAPY_fix_ and _−flex_ do not comprise therapeutic guidance in an Internet-based self-help intervention sense of way (31). Therapists are requested to check patients online-session activities prior to the next online or standard session. This process is reimbursed at 20 € within the PNP contract per therapists' check of patients' online-session activities. It is possible to provide a written feedback within the eSano platform, however, the PNP billing code is not designed for this therapeutic intervention guidance, but rather a quality assurance check of estimated 5–15 min time per patient and online session.

### Sample Size and Power Calculation

The sample size calculation is based on a random intercept model comparing the primary outcome (PHQ-ADS at T3) between treatment conditions while accounting for the nested structure of the data. Although this model is simpler than the target statistical analyses for the primary outcome presented below, it allows to avoid speculative assumptions about numerous unknown model parameters. The focal hypothesis is that both intervention groups PSYCHOnlineTHERAPY_fix/flex_ are not inferior to CBT_standard_ (= non-inferiority trial). Non-inferiority is assumed if the confidence interval (CI) of the standardized mean comparissson between PSYCHOnlineTHERAPY_fix/flex_ is completely above SMD = −0.24, which is considered as a lower threshold of clinical significance (49). We assume one-sided tests with α = 0.025 (Bonferroni-adjusted) and 1-ß = 0.8, an intra-cluster correlation coefficient (ICC) of 0.01 with a median cluster size of 12 eligible patients as feasible number to be recruited within the recruitment period. Based on these assumptions, the present trial aims at a sample size of 25 psychotherapy outpatient practice clusters and *n* = 300 patients per study arm with an allocation ratio of 1:1:1 (PSYCHOnlineTHERAPY_fix_, PSYCHOnlineTHERAPY_flex_, CBT_standard_; for formulas see (50).

### Assessments

All assessments will be conducted online (patient and therapist self-reports) or telephone-based (standardized clinical interview SCID-5, HAM-A, QIDS-C). For an overview of instruments at baseline (T0), inter-session assessments at six (T1) and 12 weeks (T2) follow-up, as well as 18 weeks (T3; assumed as post-treatment), 24 weeks (T4) and 12 months post-inclusion follow-up (T5) see Tables 3, 4. PSYCHOnlineTHERAPY might be continued with further follow-up assessments (2–5-year follow-up assessments) in case of patients' informed consent and given follow-up assessment resources.

**Table 3:** Overview of the assessments (patients).

**Variable**	**Instrument**	**Time of measurement**					
		**T0**	**T1**	**T2**	**T3**	**T4**	**T5**
**Online-questionnaires (self-rated)**							
Depression and anxiety	PHQ-ADS	X	X	X	X	X	X
Quality of Life	AQOL	X			X	X	
Patient satisfaction	ZUF-8				X		
Working alliance	WAI-SR		X		X		
Cost-effectiveness	TIC-P	X			X	X	
Sociodemographics	SR	X	X				
Risk factors	SR	X					
Medication	SR	X			X	X	X
Reasons for dropout	SR				X		
Childhood trauma	CTS	X					
Social support	F-SozU K-6	X			X		
Negative effects	NEQ				X		
Suicidal and self- injurious thoughts and behaviors	C-SSRS	X			X		
Personality functioning	LPFS-BF 2.0	X					
Self-care	SR	X	X	X	X		
Self-management	SMST	X	X	X	X		
Self-efficacy	MHSES	X	X	X	X		
Therapeutic agency	TAI		X	X	X		
Empowerment	SR				X		
CBT-Skills	CBTSQ	X	X	X	X		
Homework implementation	SR		X	X	X		
Loneliness	UCLA	X			X		
Expectations/Credibility	CEQ	X			X		
Attitudes toward blended therapy	APOI	X			X		
Individual therapy goals	FRAPT	X			X		
**Interviews (clinician-rated)**							
Comorbid disorders, severity, chronicity; remission	SCID-5	X			X		
Depression response	QIDS	X			X		
Anxiety response	HAM-A	X			X		
Serious adverse events	Checklist				X		

*T0, Baseline; T1, 6 weeks; T2, 12 weeks; T3, 18 weeks post inclusion; T4, 24 weeks post inclusion; T5, 12 months post inclusion follow-up; PHQ-ADS, Patient Health Questionnaire Anxiety and Depression Scale; AQOL, Assessment of Quality of Life; ZUF-8, Questionnaire for Patient Satisfaction-8; WAI-SR, Working Alliance Inventory—Short Revised; TIC-P, Trimbos Institute and Institute of Medical Technology Questionnaire for Costs Associated with Psychiatric Illness; SR, Self-Report Assessment; CTS, Childhood Trauma Screener; F-SozU K-6, Perceived Social Support Questionnaire brief form; NEQ, Negative Effects Questionnaire; C-SSRS, Columbia-Suicide Severity Rating Scale; LPFS-BF 2.0, Level of Personality Functioning Scale Brief Form 2.0; SMST, Self-Management Self-Test; MHSES, Mental Health Self Efficacy Scale; TAI, Therapeutic Agency Inventory; CBTSQ, Cognitive-Behavioral Therapy Skills Questionnaire; UCLA, UCLA Three-Item Loneliness Scale; CEQ, Credibility-Expectancy-Questionnaire; APOI, Attitudes toward Psychological Online Interventions; FRAPT, Fragebogen zur Messung persönlicher Therapieziele [Questionnaire for measuring personal therapy goals]; SCID-5, Structured Clinical Interview for DSM-5; QIDS, Quick Inventory of depressive Symptomatology; HAM-A, Hamilton Anxiety Rating Scale*.

**Table 4:** Overview of the assessments (therapists).

**Variable**	**Instrument**	**Time of measurement**					
		**T0**	**T1**	**T2**	**T3**	**T4**	**T5**
Working alliance	WAI-SR-T				X		
Sociodemographics	SR	X					
Attitudes toward blended therapy	APOI	X			X^*^		
Attitudes toward evidence-based practice	EBPAS-D36	X					
Willingness to use digital health interventions	SR	X					
Barriers/facilitators in the use of digital health interventions	SR	X					
Acceptance of components of blended therapy	SR	X					
Determinants of behavioral change	TDF				X^*^		
Degree of normalization	NoMAD				X^*^		

*T0, Baseline; T1, 6 weeks; T2, 12 weeks; T3, 18 weeks post inclusion; T4, 24 weeks post inclusion; T5, 12 months post inclusion follow-up; WAI-SR-T, Working Alliance Inventory—Short Revised therapist version; SR, Self-Report Assessment; APOI, Attitudes toward Psychological Online Interventions; EBPAS-D36, Evidence-based Practice Attitude Scale-36; TDF, Theoretical Domain Framework Questionnaire; NoMAD, Normalization Measure Development Questionnaire;*

* *Assessment at the end of the entire study*.

#### Primary Outcome

The primary outcome is depression and anxiety severity at post-treatment 18 weeks post-inclusion (T3), assessed with the Patient Health Questionnaire Anxiety and Depression Scale [PHQ-ADS; 16 items, score range: 0–48; (51)]. Depression and anxiety symptoms at all other assessments will be considered as secondary outcomes. PHQ-ADS is the combined sum score of the questionnaires Generalized Anxiety Disorder Screener (GAD-7) and Patient Health Questionnaire (PHQ-9) as a composite measure of depression and anxiety, with good internal consistency [α = 0.88–0.92; (51)].

#### Secondary Outcomes

Depression and anxiety remission will be assessed with the Structured Clinical Interview [SCID-5; (52)] as a comprehensive, structured interview designed to be used by trained interviewers for the assessment of mental disorders according to the definitions and criteria of DSM-5. It enables a reliable, valid, and efficient assessment of depressive disorders (52). The SCID-5 will also be used in order to obtain additional information about comorbid disorders, severity of disorders, and chronicity.

Depression response will be assessed with the Quick Inventory of Depressive Symptomatology in its clinician-rated version [QIDS-C; 16 items, score range: 0–27; (53)]. It encompasses the criteria sleep, depressed mood, appetite/weight change, concentration/decision making, self-outlook, suicidal ideation, loss of interest or pleasure, energy/fatigability, and psychomotor changes. QIDS depressive symptom scores are used to determine depression response in accordance to the recommendations of Jacobson and Truax (54). Trained interviewers will conduct the clinician-rated version QIDS-C for which good psychometric properties and internal consistencies between α = 0.81 and α = 0.95 are reported (55).

Anxiety response will be assessed with the Hamilton Anxiety Rating Scale [HAM-A; 14 items, score range: 0–56; (56, 57)] which measures psychic and somatic symptoms of anxiety. Like the QIDS-C and the SCID-5, the HAM-A is clinician-rated and therefore will be conducted by a trained interviewer. It is characterized by a high inter-rater reliability and internal consistency (α = 0.85; 57). HAM-A anxiety symptom scores are used to determine anxiety response in accordance to the recommendations of Jacobson and Truax (54).

Health-related quality of life will be assessed with the self-report questionnaire Assessment of Quality of Life [AQoL-8D; 35 items; (58, 59)] including the unweighted responses subscales physical super-dimension (range: 10–51) and psychosocial/mental super-dimension (range: 25–125) and a total score (range: 35–176). The AQoL-8D is characterized by a high Cronbach's Alpha of 0.96 and good psychometric properties (58).

Patient satisfaction will be assessed with a German short version [ZUF-8; eight items, score range: 8–32; (60)] of the Client Satisfaction Questionnaire [CSQ; (61)]. Higher scores are indicative for higher satisfaction. Internal consistency of the ZUF-8 is reported with a Cronbach's Alpha of 0.90 (60). In addition, reasons for dissatisfaction with the intervention will be assessed with 9 self-developed items.

Working alliance will be assessed with the German version (62) of the Working Alliance Inventory [WAI-SR; 12 items, score range: 12–60; (63)]. It covers the three subscales (a) agreement on tasks (four items), (b) agreement on goals (four items), and (c) development of an affective bond (four items). For the German Version, internal consistencies between α = 0.81 and α = 0.91 were reported for the subscales and internal consistencies between α = 0.90 and α = 0.93 for the total score (62, 64). Participants will complete the WAI-SR at T1 and at T3. Therapists only at T3. This will allow for a comparison between patients' and therapists' view on working alliance.

Psychotherapy adherence will be assessed by means of the number of completed online- and standard sessions. Per-protocol (PP) adherence is operationalized by the percentage of participants that completed their psychotherapy as recommended by their therapist (therapist-rating). Reasons for dropout are assessed by six items at post-treatment (T3; patient-rating).

#### Covariates

As potential moderating variables, demographic (e.g., gender, age, education) and medical information (e.g., previous treatment, medication) will be recorded at baseline. Further, a variety of potential predictors will be included to assess moderators and mediators of psychotherapy effects.

The following information of therapists will be assessed with six items: age, gender, time since licensed as therapist, number of inhabitants at the location of the practice, and experience with digitally supported psychotherapy. Patient characteristics will be assessed with 15 self-report items including information on age, gender, body height, weight, education, employment, income, relationship status, children living in the household, ethnicity, migration, previous treatment, number of inhabitants of the place of residence, and distance to the practice of the therapist.

Further patient characteristics (risk factors) that potentially predict depression and anxiety symptoms will be assessed by means of 27 self-report items. The following factors will be assessed: smoking, drug use, alcohol consumption, diet quality, social status, minority, discrimination, self-perceived energy, family history of mental illness, adverse childhood experiences (parental death or divorce), accidents, physical and sexual abuse, and physical activity.

Existing medication at the beginning of the study will be assessed at baseline (three items). Potential initiation of new medication will be assessed at post-treatment (two items) and at follow-ups (three items).

Childhood trauma will be assessed with the Childhood Trauma Screener [CTS; five items, score range: 5–25; (65, 66)]. Its internal consistency is reported to be α = 0.76 (65).

Social support will be assessed with the brief form of the Perceived Social Support Questionnaire [F-SozU K-6; six items, score range: 6–30; (67)]. Higher scores indicate higher perceived social support. The measure is characterized by a high Cronbach's alpha of 0.90 (67).

Personality functioning will be assessed with the German version of the Level of Personality Functioning Scale—Brief Form 2.0 [LPFS-BF 2.0; 12 items, score range: 12–48; (68)]. Personality functioning is divided into the two subscales interpersonal-functioning (six items) and self-functioning (six items). The internal consistency of the total scale is reported to be α = 0.82 (69).

Self-management will be assessed with the Self-Management Self-Test (SMST; five items, score range: 0–20; 70). Higher scores are indicative for better self-management competence. The SMST has been shown to have good psychometric properties with a Cronbach's Alpha of 0.80 (70).

Self-efficacy will be assessed with the Mental Health Self Efficacy Scale [MHSES; six items, score range: 6–60; (71)]. The instrument shows a Cronbach's Alpha of 0.89 (71).

Self-care will be assessed by means of four items (score range: 0–34) that were newly developed for this study. They consist of statements about having and taking time, personal resources for oneself, and the engagement with therapy contents at home.

Therapeutic agency will be assessed with two selected subscales of the Therapeutic Agency Inventory [TAI; 10 items; (72)]. The dimensions therapy-related processing (five items, score range: 1–5) and therapist-oriented passivity (five items, score range: 1–5) will be used within our study. Higher scores indicate higher levels of therapeutic agency. Internal consistency of the subscales is reported with α = 0.79 (therapy-related processing) and α = 0.73 [therapist-oriented passivity; (72)].

Empowerment will be assessed with two open-ended questions. Patients will be asked whether and how the intervention contributed to a feeling of strength and confidence and how they experience this in their everyday life.

CBT-skills will be assessed with the Cognitive-Behavioral Skills Questionnaire [CBTSQ; 16 items; (73)]. The measure comprises the two subscales behavioral activation (seven items, score range: 1–5) and cognitive restructuring (nine items, score range: 1–5). Higher scores are indicative of greater use of CBT-skills. Internal consistency is reported to be α = 0.88 (cognitive restructuring) and α = 0.85 [behavioral activation; (73)].

Homework implementation will be assessed with three self-developed items regarding adherence to exercises between the sessions, levels of difficulty in homework completion, and reasons for non-adherence.

Loneliness will be assessed with the UCLA Three-Item Loneliness Scale [UCLA; three items, score range: 3–9; (74)]. Higher scores are indicative for greater loneliness. It is characterized by a Cronbach's Alpha of 0.72 (74).

Expectations and credibility regarding the intervention will be assessed with the Credibility/Expectancy Questionnaire [CEQ; six items; (75, 76)]. The CEQ consists of the two distinct factors credibility (three items, score range: 3–27) and expectancy (three items, score range: 3–27). In order to re-evaluate expectancies of patients, treatment credibility will be assessed again at post-treatment (T3). Cronbach's Alpha of the total scale is ranging between α = 0.84 and α = 0.85, between α = 0.79 and α = 0.90 for the expectancy factor, and between α = 0.81 and α = 0.86 for the credibility factor (76).

Attitudes toward blended therapy will be assessed with selected subscales of the Attitudes toward Psychological Online Interventions Questionnaire [APOI; four items patients/12 items therapists; (77)] which was adapted to blended therapy for this study. For patients the subscale confidence in effectiveness (four items, score range: 1–5) will be used. From the therapist version we will use the following three subscales: skepticism and perception of risk (four items, score range: 1–5), confidence in effectiveness (four items, score range: 1–5), and technologization threat (four items, score-range: 1–5). Depending on the specific subscale higher scores indicate a more negative or positive attitude toward blended therapy. Internal consistency of the total scale is reported to be α = 0.77 with the subscales ranging from α = 0.62–α = 0.72 (77).

Individual therapy goals will be assessed with 4 selected subscales of the “Fragebogen zur Messung persönlicher Therapieziele” [Questionnaire for measuring personal therapy goals] [FRAPT; 31 items; (78)]. The subscales cover the overall goal categories trust in yourself and others (14 items, score range: 0–3), active confrontation with oneself and the disease (nine items, score range: 0–3), coping with depression and anxiety (four items, score range: 0–3), and family and improvements in the family and socioeconomic conditions (four items, score range: 0–3). Internal consistencies of the subscales are ranging from α = 0.67–α = 0.92 (78). Individual therapy goals will be assessed at T0 and their achievement will be evaluated with an adapted version of the measure at T3.

Attitudes toward evidence-based practice will be assessed with selected subscales of the German version of the Evidence-based Practice Attitude Scale-36 [EBPAS-D36; 12 items; (79)]. We will use the scales openness (three items, score range: 0–4), divergence (three items, score range: 0–4), limitations (three items, score range: 0–4), and balance (three items, score range: 0–4). For the English version Cronbach's Alpha is reported to range between α = 0.60 and α = 0.90 for the used subscales (80).

Three newly developed scales will be employed to assess therapists' willingness to use digital health interventions (six items, score range: 0–4), their experiencing of barriers and facilitators in the use of digital health interventions (eight items, score range: 0–4), and their acceptance of components of blended therapy (nine items, score range: 0–4).

Determinants of behavioral change that influence the behavior in health care settings when new interventions are implemented will be assessed with a theoretical domains framework (TDF) questionnaire [32 items; (81)] including the following dimensions: knowledge (four items, score range: 1–7), skills (three items, score range: 1–7), social/professional role and identity (four items, score range: 1–7), beliefs about capabilities (three items, score range: 1–7), optimism (two items, score range: 1–7), beliefs about consequences (two items, score range: 1–7), intentions (four items, score range: 1–7/1–10), memory, attention, and decision processes (four items, score range: 1–7), environmental context and resources (two items, score range: 1–7), social influences (two items, score range: 1–7), and emotion (four items, score range: 1–7).

The Normalization Measure Development Questionnaire [NoMAD; 20 items, score range: 0–80; (82)] assesses the extent to which the newly implemented intervention PSYCHOnlineTHERAPY is a normal part of the daily working routine of therapists. The measure is characterized by a Cronbach's Alpha of 0.89 (83).

Documentation of the therapy process will be performed by participating therapists for each of their patients via tablet at the beginning and at the end of the therapy process and after each session regarding homework assignments, topic of the session, therapeutic techniques, serious adverse events, and orientation toward individual therapy goals.

#### Mobile Sensing

Mobile sensing data will be collected via the “AWARE” framework [https://awareframework.com; (84)]. In short, the AWARE app allows the collection of data on smartphone usage (e.g., usage time and frequency, GPS data, communication behavior) as well as ecological momentary assessments in form of questionnaires. For technical details on how the data is collected as well as an in-depth description of privacy and data security of the app and the server please see the concept paper of the AWARE framework (84).

After giving consent to participate in the main trial patients are informed about the optional mobile sensing sub-study and asked whether they would like to participate. Participants that provided their additional informed consent are instructed to install the mobile application AWARE on their personal smartphones. After installation participants can choose which of the following data points are collected over the 6 months:

##### Active Data

Anxiety and depression via the four-item version of the Patient Health Questionnaire [PHQ-4; (85)], drive, sleep quality; data on the use and acceptance of mobile sensing; quality of the application from the user's perspective via the German version of the Mobile Application Rating Scale [MARS-G; (86, 87)].

##### Passive Data

Duration and frequency of smartphone usage, calls, and SMS; number of words in SMS; usage duration and frequency of installed apps, keyboard input, GPS, type of movement, other movement information (acceleration, rotation, gravity), battery status, screen status, phone events, ambient light, ambient noise, and weather at the location.

#### Side Effects and Adverse Events

We include different ways of monitoring and assessing side effects and (serious) adverse events [(S)AEs] adapted from the National Institute for Health Research recommendations (88) and Horigian et al. (89) who give general principles to define (S)AEs.

We define AEs as adverse or unintended symptoms or conditions that are inconsistent in nature or severity with the present information about the effects of the intervention. SAEs include the following events: (1) emergency hospitalization due to mental illness, (2) breakdown of a close, important relationship, (3) intoxication with a psychotropic substance requiring medical care, (4) self-injury (intentional) requiring medical care, (5) suicide or suicide attempt, (6) acute psychosis.

(S)AEs may be reported in telephone interviews and during psychotherapy sessions. Psychotherapists and interviewers are required to report (S)AEs to the trial evaluation administration. In addition, the possible occurrence of SAEs is systematically queried at the end of the telephone interview at T3.

Additionally, possible negative effects of the psychotherapies are assessed by means of the 20-item version of the Negative Effects Questionnaire [NEQ; 20 items; (90, 91)] which measures the frequency (score range: 0–20) and impact (score range: 0–80) of several different negative effects during the treatment period. Internal Consistency of the NEQ is reported to be α = 0.95 (90). Moreover, depression and anxiety symptom deterioration are determined by means of the QIDS-C and HAM-A.

Within the online-questionnaires suicidal and self-injurious thoughts and behaviors will be assessed by means of a modified version of the Columbia-Suicide Severity Rating Scale (92) at baseline (T0) and post-treatment (T3). At all times of measurement suicidal tendencies are measured by PHQ-ADS Item 9.

#### Key Economic Outcomes

##### Health-Related Outcomes

In the cost-effectiveness analyses, the main outcome will be response according to PHQ-ADS. In the cost-utility analysis, quality-adjusted life years (QALYs) will be the health-related outcome based on the AQoL-8D. The AQoL-8D generates patient preference–based utilities on a scale of 0 (death) to 1 (perfect health), using the time-trade-off method (93). QALY gains will be estimated by calculating the area under the curve (AUC) of linearly interpolated AQoL-8D utilities between measurement points to cover the follow-up period.

##### Cost Measures

The health-economic evaluation will be based on claims data provided by the statutory health insurance companies AOK Baden-Wuerttemberg and Bosch BKK and the German version of the Dutch cost questionnaire Trimbos Institute and Institute of Medical Technology Questionnaire for Costs Associated with Psychiatric Illness [TiC-P; (94, 95)], which was specifically adapted to the population of psychotherapy outpatients in Germany. Claims data will contain basic information on the insured persons like sex, age, and profession as well as information on costs, diagnoses, and treatments for the following areas: in- and outpatient care, rehabilitation, prescribed medication, and therapeutic appliances and remedies, as well as sickness benefits and disability pension. Each online session will be charged with 20 €. In addition, Ulm University will provide information on costs for providing the digital treatment to psychotherapists. We will use the TiC-P for collecting data on patient and family costs (e.g., out-of-pocket expenses) and productivity costs due to presenteeism (i.e., reduced efficiency while at work). Lost workdays due to presenteeism will be computed by taking into account the number of work days for which the participant reported reduced functioning weighted by the reported corresponding inefficiency score for those days (96).

#### Qualitative Semi-Structured Interviews

Qualitative semi-structured interviews will be conducted with a sub-group of psychotherapists in a focus group setting and with patients in individual interviews. Trained interviewers will explore acceptance, usage behavior, barriers, and facilitators of PSYCHOnlineTHERAPY_fix/flex_. The interview-guides will be developed theory-based after a literature review and with the involvement of experts.

A qualitative method with a theory-based approach should gain insights into the perspectives and experiences of both participant groups. Hence, interviews with psychotherapists will investigate barriers to and facilitators for the implementation of blended psychotherapy for depression and/or anxiety. The interview guide for psychotherapists will take into account the TDF (97), that aims to identify domains that influence the implementation of interventions and professionals‘behavior change.

Interviews with patients will provide insights into the participants' experience (e.g., acceptance, feasibility of intervention usage) with the Internet-based interventions which are blended with face-to-face sessions. The interview guide will take into account the Unified Theory of Acceptance and Use of Technology (UTAUT) model (98).

The sample size and composition will be planned to consider the different intervention groups and gain sufficient theoretical data saturation. Both PSYCHOnlineTHERAPY_fix_ and _−flex_ will be represented. Based on sample size guidelines (99), ~20 participants per group (PSYCHOnlineTHERAPY_fix/flex_) in each study with psychotherapists and patients are estimated to be necessary. However, final sampling follows theoretical data saturation principles (100, 101).

#### Reimbursement

Trial participants will receive the following compensations for their study related efforts:

Trial psychotherapists will receive 1.000 € for the one-day training course as compensation for their non-realized incomes, 44.80 € for each successfully recruited study patient as compensation for the time necessary to conduct the informed consent process, and 120 € for every provided complete therapy process documentation sheet per patient after completion of the treatment within the study. Additionally, psychotherapists taking part in the qualitative interview will receive 100 € as compensation for their effort and not-realized income in this time.

Trial patients will receive 20 € for completed 18 weeks (T3) and 6 months (T4) follow-up. T5 onwards are not covered by the PSYCHOnlineTHERAPY grant. Hence, compensation can only be realized in case of additional funding. Additionally, patients taking part in the qualitative interview will receive 30 € as compensation for their effort.

#### Data Management, Quality Assurance, and Safety Measures

All online assessments will be completed via LimeSurvey (installed on an internal server of the University of Erlangen-Nuernberg) and data entered will be transmitted directly and in pseudonymized form to the data-handling center at the University of Erlangen-Nuernberg. For each individual data assessment, an individualized link including the study ID of the participant is used. Participants will receive their individual links by email. For the management of participants in the therapy setting, a tablet with an integrated app is used for the therapy documentation which does not save any data but forwards them to LimeSurvey. The evaluating center will monitor the quality and completeness of the data and the compliance of the measurements with the assessment schedule determined by the study protocol.

#### Procedure on (S)AEs

Information about potential (S)AEs are obtained within diagnostic interviews, online questionnaires, and during therapy sessions. Whenever a (S)AE is detected, the incidence will be documented by either the therapist or the study personnel. During the treatment period especially the therapist is responsible for dealing with occurring (S)AEs and is informed by the study personnel about (S)AEs identified through answers of patients within the interviews or questionnaires. Critical answers within interviews are identified and documented by the interviewers who are trained in recognizing (S)AEs and provided with a detailed instruction in dealing with suicidality during interviews. Critical answers within questionnaires as defined by a score >1 on PHQ-ADS Item 9 will be forwarded to the therapist. Therapists will have to document (S)AEs and inform the study administration within 48 h on weekdays (SAEs) or within 1 week (AEs). Events occurring during the follow-up period are documented and handled by the study personnel by automatically sending information on help and emergency numbers and offering a meeting appointment via telephone for patients who give critical answers within the interviews or questionnaires. Critical answers as defined as a score >0 on PHQ-ADS Item 9 and selected items of our modified version of the Columbia-Suicide Severity Rating Scale will lead to automatic messages to patients with information on help and emergency numbers at all times of measurement. Documentations of (S)AEs will be forwarded to an independent Data Safety Monitoring Board (DSMB) which will monitor the frequency and severity of the occurring (S)AEs. Every 6 months the DSMB will be informed about documented (S)AEs and the recruitment process and if necessary can give recommendations for discontinuation or modification of the study.

### Statistical Analyses

#### Clinical Analyses

The primary outcome will be analyzed using a three-level random slope model with measurement points nested in patients and patients nested in therapists. We will include data on depression and anxiety severity assessed at four measurement points, including baseline (T0), inter-session assessments at six (T1) and 12 weeks (T2) follow-up, and post-treatment at 18 weeks post-inclusion (T3). Depression and anxiety severity will be predicted from log-transformed time (i.e., weeks since baseline), dummy-coded treatment groups, and their interactions. Random intercepts and slopes will be included to account for unexplained heterogeneity in baseline status and rate of change, both at the level of patients and therapists. The focal tests of non-inferiority will be based on the bootstrap CIs of the interactions between time and groups. All statistical analyses will be performed based on intention-to-treat (ITT) principle. Patterns of missing data will be examined and analyses will be corrected for missing data by applying multiple imputation. Additional multiple PP analyses will be conducted including only patients that provided data.

#### Economic Evaluation

The economic evaluation will be performed from a societal and a public health care perspective. Two multilevel models (MLMs) will be specified, one for costs and one for effects, which consider the hierarchical structure of the data. For effects, normal-based 95% CIs will be estimated. For costs, 95% CIs will be estimated using bias-corrected and accelerated (BCa) bootstrapping with 5,000 replications (102). MLMs will be combined with cluster bootstrapping, which is recommended for resampling clustered data (103). Across the three treatment groups, the adjusted mean costs and QALYs will be compared to assess if any of the treatments are less effective and more expensive than the other treatments. If so, incremental cost-effectiveness ratios (ICERs) will not be estimated in relation to that treatment (104). Otherwise, ICERs will be estimated by calculating the difference in costs between two treatment options divided by the difference in effectiveness of these two treatment options. The joint uncertainty surrounding costs and effects will be summarized using cost-effectiveness acceptability curves [CEACs; (105)]. CEACs show the probability of an intervention being cost effective in comparison with the alternatives for a range of different ceiling ratios.

#### Moderator Analyses

Predictors and moderators of treatment outcome will be analyzed on an exploratory basis with a priori defined potential moderators. We will conduct univariate exploratory analyses by entering the respective baseline variable as a three-way interaction term in the three-level random slope model. Moreover, we will also investigate interindividual differences in treatment effects by utilizing the EffectLiteR approach (106), that allows to include interactions between the treatment variable and a range of categorical and continuous (latent) covariates, because it uses a multigroup structural equation model for the estimation of parameters.

#### Mobile Sensing

The analysis of the mobile sensing data will be divided into two parts. Firstly, correlation analyses will be conducted to investigate associations between digital features (e.g., smartphone usage time) and mental health outcomes. Correlation coefficient r will be used for the analysis, which ranges between 0 (no relationship) to 1 (perfect relationship; −1 perfect negative relationship). For all correlation analyses, the alpha-level will be 5%. *P*-values will be adjusted for multiple testing using the procedure proposed by Holm (107, 108). Full information maximum likelihood will be applied to deal with missing values in the correlation analysis (108, 109). Secondly, we will build different prediction models to predict mental health. Both “traditional” multilevel models relying on significance tests and machine learning will be used. Building and modifying machine learning models is a highly iterative process with many changing factors to find the optimal model (e.g., architecture, optimizer, included features). Hence, defining a-priori model is impossible. However, a range of approaches will be tested, this includes: Random Forest models, Support Vector Machine (SVM), XGBoost (XGB), K-Nearest Neighbor (KNN), and Logistic Regression [LR; (110–114)].

#### Qualitative Data Analysis

All qualitative data will be audiotaped and transcribed verbatim using the software MAXQDA. The analysis of qualitative data will be based on qualitative content analysis. An inductive-deductive approach will be applied linked to the theory-based interview guide. To establish reliability of results (indicated by intercoder agreement), two independent raters will code all transcripts on the basis of coding guide and rules. The development of this coding guide follows an iterative process with consensus finding. A follow-up survey with the interviewed samples enable the validation of emergent themes. A higher representativity of the results should enable the subsequent validation of the identified themes by a survey with all participants. Meaningful differences in the identified themes between the two groups PSYCHOnlineTHERAPY_fix_ and _−flex_ will be described through the report of group differences of at least 25%.

## Discussion

PSYCHOnlineTHERAPY will be one of the largest ever-conducted psychotherapy trials with 900 patients from 75 psychotherapy standard outpatient care practices. At the same time PSYCHOnlineTHERAPY has the potential of innovating psychotherapy in the near future by extending the ways of conducting psychotherapy on-site, video-conference based, Internet-and mobile-based, or blended using potentially all of these possibilities as examined in PSYCHOnlineTHERAPY. The rigorous health care services approach of PSYCHOnlineTHERAPY, embedded into standard care with even new billing codes as part of the underlying PNP contract for the new psychotherapeutic service of the Internet- and mobile-based intervention modules will ensure the timely implementation of blended therapy into standard care, in case of PSYCHOnlineTHERAPY being effective and cost-effective. Moreover, the elaborated evaluation concept on moderators and mediators including a smart sensing sub-study, will provide deeper insights into the black box psychotherapy, i.e., the active components and the mechanisms of change (32, 115) as well as the differential indication question on which type of psychotherapy and specific technique works for whom at what time in the treatment course (9, 116–118).

### Potential Problems and Solutions

In order to avoid selection bias, PSYCHOnlineTHERAPY follows strict trial role division with Ulm University as principle investigator, intervention content and IT-solution provider and interface to health care service providers and stakeholders. Evaluation of PSYCHOnlineTHERAPY takes place at University of Erlangen-Nuernberg. Thereby, the University of Erlangen-Nuernberg has established a work flow ensuring that randomization, administration of participants, and outcome assessments (interviews) will be conducted independent of each other. Particularly outcome assessors are kept blind toward trial arm allocation. At the beginning of each telephone interview, patients are asked to keep their trial arm allocation disclosed.

Trial arm contamination might occur in joint practices. Hence, psychotherapists in joint practices are randomized and allocated jointly in the same trial arm condition. Resulting clustering effects will be compensated statistically. Different to cRCTs in general practices patient-level trial arm contamination is rather unlikely, as patients do not switch psychotherapists in due course or during times of absence (e.g., vacation) of their psychotherapists. Patients will be asked whether they already took part in PSYCHOnlineTHERAPY before.

Considering that therapeutic alliance, which depends also on the characteristics of the psychotherapists, is one of the most important variables predicting the outcome of psychotherapy (119, 120), randomizing therapists runs the risk of clinically important baseline imbalance. Cluster size was optimized for the pre-specified non-inferiority margin, however, between trial arm baseline imbalance regarding therapist competency needs to be considered carefully when interpreting the final trial results.

Study and intervention protocol adherence will be improved by structured training courses, regular contacts with study psychotherapists as well as assessed via therapists' documentation of their psychotherapies.

## Conclusion

The present cRCT PSYCHOnlineTHERAPY aims to examine the non-inferiority and cost-effectiveness of two blended therapy versions embedded in standard care. As one of the largest psychotherapy trials ever conducted, PSYCHOnlineTHERAPY has the potential to innovate the way of how psychotherapy is provided, thereby exploring its mechanisms of change at the same time.

## Trial Status

Patient Recruitment start is scheduled for January 2021.

## Dissemination

Trial results will be presented on national and international conferences and published in peer-reviewed journals. Access to the final trial dataset can be provided on request depending on to be specified data security and data exchange regulation agreements.

## Ethics Statement

The studies involving human participants were reviewed and approved by Ethics Committee of the German Asssociation of Psychology. The patients/participants provided their written informed consent to participate in this study.

## Author Contributions

HB, DDE, MR, and RP, MEDIVERBUND AG, AOK Baden-Wuerttemberg, and Bosch BKK obtained funding for this study. HB, NB, A-CZ, LB, CH, PM, TS, MS, LSteu, YT, IT, and DE contributed to the study design. ARI, RK, TN, RP, MR, and LSten developed the platform eSano and contributed to the technical study design. CB contributed to the design of the health-economic evaluation. HB drafted the manuscript and is principle investigator of PSYCHOnlineTHERAPY. All authors contributed to the article and approved the submitted version.

## Conflict of Interest

Authors at Ulm University were partly involved in the development of PSYCHOnlineTHERAPY. Therefore, evaluation of the trial will be independently conducted by the evaluator at University of Erlangen-Nuernberg. HB received consultancy fees, reimbursement of congress attendance, and travel costs as well as payments for lectures from Psychotherapy and Psychiatry Associations as well as Psychotherapy Training Institutes in the context of E-Mental-Health topics. He has been the beneficiary of study support (third-party funding) from several public funding organizations. DE possesses shares in the GET.On Institut GmbH (HelloBetter), which works to transfer research findings on IMIs into standard care. DE has received payments from several companies and health insurance providers for advice on the use of IMIs. He has received payments for lectures from Psychotherapy and Psychiatry Associations and has been the beneficiary of third-party funding from health insurance providers. IT has received fees and travel costs for lectures or workshops in the eHealth setting from congresses and psychotherapy training institutes. The remaining authors declare that the research was conducted in the absence of any commercial or financial relationships that could be construed as a potential conflict of interest.

## Abbreviations

APOI: Attitudes toward Psychological Online Interventions Questionnaire
AUC: area under the curve
AQoL-8D: Assessment of Quality of Life
BCa: bias-corrected and accelerated
CBT: cognitive behavioral therapy
CBTSQ: Cognitive-Behavioral Skills Questionnaire
CEAC: cost-effectiveness acceptability curve
CEQ: Credibility/Expectancy Questionnaire
CI: confidence interval
cRCT: Cluster-randomized controlled trial
CSQ: Client Satisfaction Questionnaire
CTS: Childhood Trauma Screener
CONSORT: Consolidated Standards of Reporting Trials
COREQ: Consolidated Criteria for Reporting Qualitative Studies
DSMB: Data Safety Monitoring Board
EBPAS-D36: German version of the Evidence-based Practice Attitude Scale-36
FRAPT: “Fragebogen zur Messung persönlicher Therapieziele”
F-SozU K-6: Brief form of the Perceived Social Support Questionnaire
GAD-7: Generalized Anxiety Disorder Screener
HAM-A: Hamilton Anxiety Rating Scale
ICC: Intra-Cluster Correlation Coefficient
ICER: Incremental cost-effectiveness ratio
ITT: Intention-to-treat
IMI: Internet- and mobile-based intervention
KNN: K-Nearest Neighbor
LPFS-BF 2.0: Level of Personality Functioning Scale—Brief Form 2.0
LR: Logistic Regression
MARS-G: German version of the Mobile Application Rating Scale
MDR: Medical Device Regulation
MHSES: Mental Health Self Efficacy Scale
MLM: multilevel model
NEQ: Negative Effects Questionnaire
NoMAD: Normalization Measure Development Questionnaire
PIC/S: Pharmaceutical Inspection Cooperation Scheme
PHQ-4/-9: Patient Health Questionnaire
PHQ-ADS: Patient Health Questionnaire Anxiety and Depression Scale
PNP: Psychotherapy, Neurology, Psychosomatic and Psychiatry (selective health care services contract)
PP: Per-protocol
QALY: Quality adjusted life year
QIDS-C: Quick Inventory of Depressive Symptomatology in its clinician-rated version
(S)AE: (serious) adverse event
SCID-5: Structured Clinical Interview for DSM-5 Axis I Disorders
SMST: Self-Management Self-Test
SVM: Support Vector Machine
TAI: Therapeutic Agency Inventory
TAU: Treatment as usual
TiC-P: Trimbos Institute and Institute of Medical Technology Questionnaire for Costs Associated with Psychiatric Illness
TDF: theoretical domains framework
UCLA: UCLA Three-Item Loneliness Scale
UTAUT: Unified Theory of Acceptance and Use of Technology
WAI-SR: Working Alliance Inventory - Short Revised
XGB: XGBoost
ZUF-8: German Version of the CSQ.

## References

[1] Cuijpers P Sijbrandij M Koole SL Andersson G Beekman AT Reynolds CF. The efficacy of psychotherapy and pharmacotherapy in treating depressive and anxiety disorders: a meta-analysis of direct comparisons. World Psychiatry. (2013) 12:137–48. 10.1002/wps.20038 23737423 PMC3683266

[2] Huhn M Tardy M Spineli LM Kissling W Förstl H Pitschel-Walz G. Efficacy of pharmacotherapy and psychotherapy for adult psychiatric disorders: a systematic overview of meta-analyses. JAMA Psychiatry. (2014) 71:706–15. 10.1001/jamapsychiatry.2014.112 24789675

[3] Mack S Jacobi F Gerschler A Strehle J Höfler M Busch MA. Self-reported utilization of mental health services in the adult German population - evidence for unmet needs? Results of the DEGS1-Mental health module (DEGS1-MH). Int J Methods Psychiatr Res. (2014) 23:289–303. 10.1002/mpr.1438 24687693 PMC6878535

[4] Baldwin DS Anderson IM Nutt DJ Allgulander C Bandelow B Den Boer JA. Evidence-based pharmacological treatment of anxiety disorders, post-traumatic stress disorder and obsessive-compulsive disorder: a revision of the 2005 guidelines from the British Association for Psychopharmacology. J Psychopharmacol. (2014) 28:403–39. 10.1177/0269881114525674 24713617

[5] DGPPN BÄK KBV AWMF AkdÄ BPtK. S3-Leitlinie/Nationale Versorgungsleitlinie Unipolare Depression-Langfassung. 1st ed. Berlin: DGPPN, ÄZQ, AWMF (2009).

[6] Excellence National Institute for Health & Clinical. Depression - The Treatment and Management of Depression in Adults (Updated Edition): National Clinical Practice Guideline. Leicester: The Brititish Psychological Society & The Royal College of Psychiatrists (2010).

[7] Nübling R Bär T Jeschke K Ochs M Sarubin N Schmidt J. Versorgung psychisch kranker Erwachsener in Deutschland - Bedarf und Inanspruchnahme sowie Effektivität und Effizienz von Psychotherapie. Psychotherapeuten. (2014) 4:389–97.

[8] Ebert DD Van Daele T Nordgreen T Karekla M Compare A Zarbo C. Internet- and mobile-based psychological interventions: applications, efficacy, and potential for improving mental health: a report of the EFPA E-health taskforce. Eur Psychol. (2018) 23:167–87. 10.1027/1016-9040/a000318

[9] Messner E-M Probst T O'Rourke T Stoyanov S Baumeister H. mHealth applications: potentials, limitations, current quality and future directions. In: Baumeister H Montag C editors. Digital Phenotyping and Mobile Sensing: New Developments in Psychoinformatics. Cham: Springer (2019). p. 235–48. 10.1007/978-3-030-31620-4_15

[10] Domhardt M Letsch J Kybelka J Koenigbauer J Doebler P Baumeister H. Are Internet- and mobile-based interventions effective in adults with diagnosed panic disorder and/or agoraphobia? A systematic review and meta-analysis. J Affect Disord. (2020) 276:169–82. 10.1016/j.jad.2020.06.059 32697696

[11] Königbauer J Letsch J Doebler P Ebert DD Baumeister H. Internet- and mobile-based depression interventions for people with diagnosed depression: a systematic review and meta-analysis. J Affect Disord. (2017) 223:28–40. 10.1016/j.jad.2017.07.021 28715726

[12] Carlbring P Andersson G Cuijpers P Riper H Hedman-Lagerlöf E. Internet-based vs. face-to-face cognitive behavior therapy for psychiatric and somatic disorders: an updated systematic review and meta-analysis. Cogn Behav Ther. (2018) 47:1–18. 10.1080/16506073.2017.1401115 29215315

[13] Baumeister H Nowoczin L Lin J Seifferth H Seufert J Laubner K. Impact of an acceptance facilitating intervention on diabetes patients' acceptance of Internet-based interventions for depression: a randomized controlled trial. Diabetes Res Clin Pract. (2014) 105:30–9. 10.1016/j.diabres.2014.04.031 24862240

[14] Ebert DD Berking M Cuijpers P Lehr D Pörtner M Baumeister H. Increasing the acceptance of internet-based mental health interventions in primary care patients with depressive symptoms. a randomized controlled trial. J Affect Disord. (2015) 176:9–17. 10.1016/j.jad.2015.01.056 25682378

[15] Lamela D Cabral J Coelho S Jongenelen I. Personal stigma, determinants of intention to use technology, and acceptance of internet-based psychological interventions for depression. Int J Med Inform. (2020) 136:104076. 10.1016/j.ijmedinf.2020.104076 31962281

[16] Lin J Faust B Ebert DD Krämer L Baumeister H. A web-based acceptance-facilitating intervention for identifying patients' acceptance, uptake, and adherence of internet- and mobile-based pain interventions: randomized controlled trial. J Med Internet Res. (2018) 20:e244. 10.2196/jmir.9925 30131313 PMC6123541

[17] Baumeister H Terhorst Y Grässle C Freudenstein M Nübling R Ebert DD. Impact of an acceptance facilitating intervention on psychotherapists' acceptance of blended therapy. PLoS ONE. (2020) 15:e0236995. 10.1371/journal.pone.0236995 32785245 PMC7423074

[18] Schuster R Pokorny R Berger T Topooco N Laireiter AR. The advantages and disadvantages of online and blended therapy: survey study amongst licensed psychotherapists in Austria. J Med Internet Res. (2018) 20:e11007. 10.2196/11007 30563817 PMC6315274

[19] Baumeister H Grässle C Ebert DD Krämer LV. Blended therapy - verzahnte Psychotherapie: Das Beste aus zwei Welten? PiD - Psychother im Dialog. (2018) 19:33–8. 10.1055/a-0592-0264

[20] Erbe D Psych D Eichert HC Riper H Ebert DD. Blending face-to-face and internet-based interventions for the treatment of mental disorders in adults: systematic review. J Med Internet Res. (2017) 19:e306. 10.2196/jmir.6588 28916506 PMC5622288

[21] Kemmeren LL van Schaik A Smit JH Ruwaard J Rocha A Henriques M. Unraveling the black box: exploring usage patterns of a blended treatment for depression in a multicenter study. JMIR Ment Heal. (2019) 6:e12707. 10.2196/12707 31344670 PMC6686640

[22] Kleiboer A Smit J Bosmans J Ruwaard J Andersson G Topooco N. European COMPARative effectiveness research on blended depression treatment versus treatment-as-usual (E-COMPARED): study protocol for a randomized controlled, non-inferiority trial in eight European countries. Trials. (2016) 17:387. 10.1186/s13063-016-1511-1 27488181 PMC4972947

[23] Renn BN Hoeft TJ Lee HS Bauer AM Areán PA. Preference for in-person psychotherapy versus digital psychotherapy options for depression: survey of adults in the U.S. NPJ Digit Med. (2019) 2:1–7. 10.1038/s41746-019-0077-1 31304356 PMC6550152

[24] Titzler I Saruhanjan K Berking M Riper H Ebert DD. Barriers and facilitators for the implementation of blended psychotherapy for depression: a qualitative pilot study of therapists' perspective. Internet Interv. (2018) 12:150–64. 10.1016/j.invent.2018.01.002 30135779 PMC6096333

[25] Grünzig SD Baumeister H Bengel J Ebert D Krämer L. Effectiveness and acceptance of a web-based depression intervention during waiting time for outpatient psychotherapy: study protocol for a randomized controlled trial. Trials. (2018) 19:1–11. 10.1186/s13063-018-2657-9 29788996 PMC5964713

[26] Hennemann S Böhme K Baumeister H Bendig E Kleinstäuber M Ebert DD. Efficacy of a guided internet-based intervention (iSOMA) for somatic symptoms and related distress in university students: study protocol of a randomised controlled trial. BMJ Open. (2018) 8:24929. 10.1136/bmjopen-2018-024929 30598489 PMC6318514

[27] Kordy H Wolf M Aulich K Bürgy M Hegerl U Hüsing J. Internet-delivered disease management for recurrent depression: a multicenter randomized controlled trial. Psychother Psychosom. (2016) 85:91–8. 10.1159/000441951 26808817

[28] Sander LB Paganini S Terhorst Y Schlicker S Lin J Spanhel K. Effectiveness of a guided web-based self-help intervention to prevent depression in patients with persistent back pain: the PROD-BP randomized clinical trial. JAMA Psychiatry. (2020) 77:1001–11. 10.1001/jamapsychiatry.2020.1021 32459348 PMC7254449

[29] Berger T Krieger T Sude K Meyer B Maercker A. Evaluating an e-mental health program (“deprexis”) as adjunctive treatment tool in psychotherapy for depression: results of a pragmatic randomized controlled trial. J Affect Disord. (2018) 227:455–62. 10.1016/j.jad.2017.11.021 29154168

[30] Zwerenz R Gerzymisch K Edinger J Holme M Knickenberg RJ Spörl-Dönch S. Evaluation of an internet-based aftercare program to improve vocational reintegration after inpatient medical rehabilitation: study protocol for a cluster-randomized controlled trial. Trials. (2013) 14:26. 10.1186/1745-6215-14-26 23351836 PMC3598370

[31] Baumeister H Reichler L Munzinger M Lin J. The impact of guidance on Internet-based mental health interventions - a systematic review. Internet Interv. (2014) 1:205–15. 10.1016/j.invent.2014.08.003

[32] Domhardt M Geßlein H von Rezori RE Baumeister H. Internet- and mobile-based interventions for anxiety disorders: a meta-analytic review of intervention components. Depress Anxiety. (2019) 36:213–24. 10.1002/da.22860 30450811

[33] Messner E-M Sariyska R Mayer B Montag C Kannen C Schwerdtfeger A. Insights – future implications of passive smartphone sensing in the therapeutic context. Verhaltenstherapie. (2019) 29, 1–10. 10.1159/000501951

[34] Campbell MK Piaggio G Elbourne DR Altman DG. Consort 2010 statement: extension to cluster randomised trials. BMJ. (2012) 345:e5661. 10.1136/bmj.e5661 22951546

[35] Juszczak E Altman DG Hopewell S Schulz K. Reporting of multi-arm parallel-group randomized trials: extension of the CONSORT 2010 statement. J Am Med Assoc. (2019) 321:1610–20. 10.1001/jama.2019.3087 31012939

[36] Montgomery P Grant S Mayo-Wilson E Macdonald G Michie S Hopewell S. Reporting randomised trials of social and psychological interventions: The CONSORT-SPI 2018 extension. Trials. (2018) 19:1–14. 10.1186/s13063-018-2733-1 30060754 PMC6066921

[37] Piaggio G Elbourne DR Pocock SJ Evans SJW Altman DG. Reporting of noninferiority and equivalence randomized trials: extension of the CONSORT 2010 statement. J Am Med Assoc. (2012) 308:2594–604. 10.1001/jama.2012.87802 23268518

[38] Schulz KF Altman DG Moher D. CONSORT 2010. statement: updated guidelines for reporting parallel group randomised trials. Trials. (2010) 11:1–8. 10.1186/1745-6215-11-32 21350618 PMC3043330

[39] Zwarenstein M Treweek S Gagnier JJ Altman DG Tunis S Haynes B. Improving the reporting of pragmatic trials: an extension of the CONSORT statement. BMJ. (2008) 337:1223–6. 10.1136/bmj.a2390 19001484 PMC3266844

[40] Tong A Sainsbury P Craig J. Consolidated criteria for reporting qualitative research (COREQ): a 32-item checklist for interviews and focus groups. Int J Qual Heal Care. (2007) 19:349–57. 10.1093/intqhc/mzm042 17872937

[41] Husereau D Drummond M Petrou S Carswell C Moher D Greenberg D. Consolidated health economic evaluation reporting standards (CHEERS)-explanation and elaboration: a report of the ISPOR health economic evaluation publication guidelines good reporting practices task force. Value Heal. (2013) 16:231–50. 10.1016/j.jval.2013.02.002 23538175

[42] Ramsey SD Willke RJ Glick H Reed SD Augustovski F Jonsson B. Cost-effectiveness analysis alongside clinical trials II - An ISPOR good research practices task force report. Value Heal. (2015) 18:161–72. 10.1016/j.jval.2015.02.001 25773551

[43] Chan A-W Tetzlaff JM Altman DG Laupacis A Gøtzsche PC KrleŽa-Jerić K. SPIRIT 2013 statement: defining standard protocol items for clinical trials. Ann Intern Med. (2013) 158:200. 10.7326/0003-4819-158-3-201302050-00583 23295957 PMC5114123

[44] Robinson L Delgadillo J Kellett S. The dose-response effect in routinely delivered psychological therapies: a systematic review. Psychother Res. (2020) 30:79–96. 10.1080/10503307.2019.1566676 30661486

[45] Lin J Paganini S Sander L Lüking M Daniel Ebert D Buhrman M. An Internet-based intervention for chronic pain - a three-arm randomized controlled study of the effectiveness of guided and unguided acceptance and commitment therapy. Dtsch Arztebl Int. (2017) 114:681–8. 10.3238/arztebl.2017.0681 29082858 PMC5672594

[46] Lunkenheimer F Domhardt M Geirhos A Kilian R Mueller-Stierlin AS Holl RW. Effectiveness and cost-effectiveness of guided Internet- And mobile-based CBT for adolescents and young adults with chronic somatic conditions and comorbid depression and anxiety symptoms (youthCOACHCD): study protocol for a multicentre randomized control. Trials. (2020) 21:1–15. 10.1186/s13063-019-4041-9 32164723 PMC7069009

[47] Küchler AM Albus P Ebert DD Baumeister H. Effectiveness of an internet-based intervention for procrastination in college students (StudiCare Procrastination): study protocol of a randomized controlled trial. Internet Interv. (2019) 17:100245. 10.1016/j.invent.2019.100245 31080750 PMC6500923

[48] Baumeister H Kraft R Baumel A Pryss R Messner E-M. Persuasive E-Health design for behavior change. In: Baumeister H Montag C editors. Digital Phenotyping and Mobile Sensing: New Developments in Psychoinformatics. Cham: Springer. (2019). p. 261–76. 10.1007/978-3-030-31620-4_17

[49] Cuijpers P Turner EH Koole SL van Dijke A Smit F. What is the treshold for a clinically relevant effect? The case of major depressive disorders. Depress Anxiety. (2014) 31:374–8. 10.1002/da.22249 24677535

[50] Rutterford C Copas A Eldridge S. Methods for sample size determination in cluster randomized trials. Int J Epidemiol. (2015) 44:1051–67. 10.1093/ije/dyv113 26174515 PMC4521133

[51] Kroenke K Wu J Yu Z Bair MJ Kean J Stump T. Patient health questionnaire anxiety and depression scale: initial validation in three clinical trials. Psychosom Med. (2016) 78:716–27. 10.1097/PSY.0000000000000322 27187854 PMC4927366

[52] Beesdo-Baum K Zaudig M Wittchen H-U. SCID-5-CV: Strukturiertes Klinisches Interview für DSM-5-Störungen - Klinische Version. Göttingen: Hogrefe (2019).

[53] Rush AJ Trivedi MH Ibrahim HM Carmody TJ Arnow B Klein DN. The 16-item Quick Inventory of Depressive Symptomatology (QIDS), clinician rating (QIDS-C), and self-report (QIDS-SR): a psychometric evaluation in patients with chronic major depression. Biol Psychiatry. (2003) 54:573–83. 10.1016/S0006-3223(02)01866-8 12946886

[54] Jacobson NS Truax P. Clinical significance : a statistical approach to defining meaningful change in psychotherapy research. J Consult Clin Psychol. (1991) 59:12–9. 10.1037/0022-006X.59.1.12 2002127

[55] Trivedi MH Rush AJ Ibrahim HM Carmody TJ Biggs MM Suppes T. The Inventory of Depressive Symptomatology, Clinician Rating (IDS-C) and Self-Report (IDS-SR), and the Quick Inventory of Depressive Symptomatology, Clinician Rating (QIDS-C) and Self-Report (QIDS-SR) in public sector patients with mood disorders: a psych. Psychol Med. (2004) 34:73–82. 10.1017/S0033291703001107 14971628

[56] Hamilton M. The assessment of anxiety states by rating. Br J Med Psychol. (1959) 32:50–5. 10.1111/j.2044-8341.1959.tb00467.x 13638508

[57] Shear MK Bilt J Vander Rucci P Endicott J Lydiard B Otto MW. Reliability and validity of a structured interview guide for the Hamilton Anxiety Rating Scale (SIGH-A). Depress Anxiety. (2001) 13:166–78. 10.1002/da.1033 11413563

[58] Maxwell A Özmen M Iezzi A Richardson J. Deriving population norms for the AQoL-6D and AQoL-8D multi-attribute utility instruments from web-based data. Qual Life Res. (2016) 25:3209–19. 10.1007/s11136-016-1337-z 27344318

[59] Richardson J Iezzi A Khan MA Maxwell A. Validity and reliability of the assessment of quality of life (AQoL)-8D multi-attribute utility instrument. Patient Patient-Centered Outcomes Res. (2014) 7:85–96. 10.1007/s40271-013-0036-x 24271592 PMC3929769

[60] Kriz D Nübling R Steffanowski A Wittmann WW Schmidt J. Patientenzufriedenheit in der stationären rehabilitation: psychometrische reanalyse des ZUF-8 auf der Basis multizentrischer Stichproben verschiedener Indikation. Zeitschrift für Medizinische Psychol. (2008) 17:67–79.

[61] Attkisson CC Zwick R. The client satisfaction questionnaire. psychometric properties and correlations with service utilization and psychotherapy outcome. Eval Program Plann. (1982) 5:233–7. 10.1016/0149-7189(82)90074-X 10259963

[62] Wilmers F Munder T Leonhart R Herzog T Plassmann R Barth J. Die deutschsprachige Version des Working Alliance Inventory-short revised (WAI-SR)-Ein schulenübergreifendes, ökonomisches und empirisch validiertes Instrument zur Erfassung der therapeutischen Allianz. Klin Diagnostik und Eval. (2008) 1:343–58.

[63] Hatcher RL Gillaspy JA. Development and validation of a revised short version of the Working Alliance Inventory. Psychother Res. (2006) 16:12–25. 10.1080/10503300500352500

[64] Munder T Wilmers F Leonhart R Linster HW Barth J. Working Alliance Inventory-Short Revised (WAI-SR): psychometric properties in outpatients and inpatients. Clin Psychol Psychother. (2009) 17:231–9. 10.1002/cpp.658 20013760

[65] Grabe HJ Schulz A Schmidt CO Appel K Driessen M Wingenfeld K. Ein Screeninginstrument für Missbrauch und Vernachlässigung in der Kindheit: der Childhood Trauma Screener (CTS). Psychiatr Prax. (2012) 39:109–15. 10.1055/s-0031-1298984 22422160

[66] Glaesmer H Schulz A Häuser W Freyberger HJ Brähler E Grabe H-J. Der Childhood Trauma Screener (CTS) - Entwicklung und Validierung von Schwellenwerten zur Klassifikation. Psychiatr Prax. (2013) 40:220–6. 10.1055/s-0033-1343116 23564353

[67] Kliem S Mößle T Rehbein F Hellmann DF Zenger M Brähler E. A brief form of the Perceived Social Support Questionnaire (F-SozU) was developed, validated, and standardized. J Clin Epidemiol. (2015) 68:551–62. 10.1016/j.jclinepi.2014.11.003 25499982

[68] Zimmermann J Müller S Bach B Hutsebaut J Hummelen B Fischer F. A common metric for self-reported severity of personality disorder. Psychopathology. (2020) 53, 1–11. 10.1159/00050737732208391

[69] Weekers LC Hutsebaut J Kamphuis JH. The level of personality functioning scale-brief form 2.0: update of a brief instrument for assessing level of personality functioning. Personal Ment Health. (2019) 13:3–14. 10.1002/pmh.1434 30230242

[70] Wehmeier PM Fox T Doerr JM Schnierer N Bender M Nater UM. Development and validation of a brief measure of self-management competence: the Self-Management Self-Test (SMST). Ther Innov Regul Sci. (2019) 2168479019849879. 10.1177/2168479019849879 31303020

[71] Clarke J Proudfoot J Birch M-R Whitton AE Parker G Manicavasagar V. Effects of mental health self-efficacy on outcomes of a mobile phone and web intervention for mild-to-moderate depression, anxiety and stress: secondary analysis of a randomised controlled trial. BMC Psychiatry. (2014) 14:272. 10.1186/s12888-014-0272-1 25252853 PMC4189737

[72] Huber J Nikendei C Ehrenthal JC Schauenburg H Mander J Dinger U. Therapeutic Agency Inventory: development and psychometric validation of a patient self-report. Psychother Res. (2018) 29:919–34. 10.1080/10503307.2018.1447707 29557306

[73] Jacob KL Christopher MS Neuhaus EC. Development and validation of the cognitive-behavioral therapy skills questionnaire. Behav Modif. (2011) 35:595–618. 10.1177/0145445511419254 21893554

[74] Hughes ME Waite LJ Hawkley LC Cacioppo JT. A short scale for measuring loneliness in large surveys: results from two population-based studies. Res Aging. (2004) 26:655–72. 10.1177/0164027504268574 18504506 PMC2394670

[75] Borkovec TD Nau SD. Credibility of analogue therapy rationales. J Behav Ther Exp Psychiatry. (1972) 3:257–60. 10.1016/0005-7916(72)90045-6

[76] Devilly GJ Borkovec TD. Psychometric properties of the credibility/expectancy questionnaire. J Behav Ther Exp Psychiatry. (2000) 31:73–86. 10.1016/S0005-7916(00)00012-4 11132119

[77] Schröder J Sautier L Kriston L Berger T Meyer B Späth C. Development of a questionnaire measuring Attitudes towards Psychological Online Interventions–the APOI. J Affect Disord. (2015) 187:136–41. 10.1016/j.jad.2015.08.044 26331687

[78] Driessen M Sommer B Röstel C Malchow CP Rumpf H-J Adam B. Therapieziele in der Psychologischen Medizin - Stand der Forschung und Entwicklung eines standardisierten Instruments. Psychother Psychosom Med Psychol. (2001) 51:239–45. 10.1055/s-2001-14300 11447657

[79] Szota K Thilemann J Christiansen H Rye M Aarons GA Barke A. Validation and psychometric properties of the german version of the evidence based practice attitudes scale (EBPAS-36D). ResearchSquare. (2020). 10.21203/rs.3.rs-104485/v1 PMC8173815 34078387

[80] Rye M Torres EM Friborg O Skre I Aarons GA. The Evidence-based Practice Attitude Scale-36 (EBPAS-36): a brief and pragmatic measure of attitudes to evidence-based practice validated in US and Norwegian samples. Implement Sci. (2017) 12:44. 10.1186/s13012-017-0573-0 28372587 PMC5379724

[81] Huijg JM Gebhardt WA Crone MR Dusseldorp E Presseau J. Discriminant content validity of a theoretical domains framework questionnaire for use in implementation research. Implement Sci. (2014) 9:1–16. 10.1186/1748-5908-9-11 24423394 PMC3896680

[82] Finch TL Girling M May CR Mair FS Murray E Treweek S. NoMAD: Implementation measure based on Normalization Process Theory [Measurement instrument] (German version © 2018 by the ImpleMentAll partners) [Internet] (2015). Available online at: http://www.normalizationprocess.org (accessed April 27, 2021).

[83] Finch TL Girling M May CR Mair FS Murray E Treweek S. Improving the normalization of complex interventions: Part 2 - Validation of the NoMAD instrument for assessing implementation work based on normalization process theory (NPT) 17 Psychology and Cognitive Sciences 1701. psychology. BMC Med Res Methodol. (2018) 18:1–13. 10.1186/s12874-018-0590-y 30442094 PMC6238372

[84] Ferreira D Kostakos V Dey AK. AWARE: mobile context instrumentation framework. Front ICT. (2015) 2:6. 10.3389/fict.2015.00006

[85] Kroenke K Spitzer RL Williams JBW Löwe B. An ultra-brief screening scale for anxiety and depression: the PHQ−4. Psychosomatics. (2009) 50:613–21. 10.1016/S0033-3182(09)70864-3 19996233

[86] Stoyanov SR Hides L Kavanagh DJ Wilson H. Development and validation of the User Version of the Mobile Application Rating Scale (uMARS). JMIR mHealth uHealth. (2016) 4:e72. 10.2196/mhealth.5849 27287964 PMC4920963

[87] Messner EM Terhorst Y Barke A Baumeister H Stoyanov S Hides L. The german version of the mobile app rating scale (MARS-G): development and validation study. J Med Internet Res. (2020) 22:e14479. 10.2196/14479 32217504 PMC7148545

[88] Duggan C Parry G McMurran M Davidson K Dennis J. The recording of adverse events from psychological treatments in clinical trials: evidence from a review of NIHR-funded trials. Trials. (2014) 15:1–9. 10.1186/1745-6215-15-335 25158932 PMC4152561

[89] Horigian VE Robbins MS Dominguez R Ucha J Rosa CL. Principles for defining adverse events in behavioral intervention research: lessons from a family-focused adolescent drug abuse trial. Clin Trials. (2010) 7:58–68. 10.1177/1740774509356575 20156957 PMC3163837

[90] Rozental A Kottorp A Boettcher J Andersson G Carlbring P. Negative effects of psychological treatments: an exploratory factor analysis of the negative effects questionnaire for monitoring and reporting adverse and unwanted events. PLoS ONE. (2016) 11:e0157503. 10.1371/journal.pone.0157503 27331907 PMC4917117

[91] Rozental A Kottorp A Forsström D Månsson K Boettcher J Andersson G. The Negative Effects Questionnaire: psychometric properties of an instrument for assessing negative effects in psychological treatments. Behav Cogn Psychother. (2019) 47:559–72. 10.1017/S1352465819000018 30871650

[92] Posner K Brown GK Stanley B Brent DA Yershova KV Oquendo MA. The Columbia-suicide severity rating scale: initial validity and internal consistency findings from three multisite studies with adolescents and adults. Am J Psychiatry. (2011) 168:1266–77. 10.1176/appi.ajp.2011.10111704 22193671 PMC3893686

[93] Richardson J Sinha K Iezzi A Khan MA. Modelling utility weights for the Assessment of Quality of Life (AQoL)-8D. Qual life Res. (2014) 23:2395–404. 10.1007/s11136-014-0686-8 24719017

[94] Hakkaart-van Roijen L Van Straten A Donker M Tiemens B. Manual Trimbos/iMTA questionnaire for Costs associated with Psychiatric illness (TiC-P). Rotterdam: Inst Med Technol Assessment, Erasmus Univ Rotterdam (2002).

[95] Bouwmans C De Jong K Timman R Zijlstra-Vlasveld M Van Der Feltz-Cornelis C Tan SS. Feasibility, reliability and validity of a questionnaire on healthcare consumption and productivity loss in patients with a psychiatric disorder (TiC-P). BMC Health Serv Res. (2013) 13:1–9. 10.1186/1472-6963-13-217 23768141 PMC3694473

[96] Osterhaus JT Gutterman DL Plachetka JR. Healthcare resource and lost labour costs of migraine headache in the US. Pharmacoeconomics. (1992) 2:67–76. 10.2165/00019053-199202010-00008 10146980

[97] Cane J O'Connor D Michie S. Validation of the theoretical domains framework for use in behaviour change and implementation research. Implement Sci. (2012) 7:1–17. 10.1186/1748-5908-7-37 22530986 PMC3483008

[98] Venkatesh V Morris MG Davis GB Davis FD. User acceptance of information technology: Toward a unified view. MIS Q Manag Inf Syst. (2003) 27:425–78. 10.2307/30036540

[99] Creswell J. Qualitative Inquiry and Research Design: Choosing Among Five Approaches. Thousand Oaks, CA: Sage Publications (1998).

[100] Aldiabat KM Le Navenec C-L. Data saturation: the mysterious step in grounded theory methodology. Qual Rep. (2018) 23:245–61. 10.46743/2160-3715/2018.2994

[101] Francis JJ Johnston M Robertson C Glidewell L Entwistle V Eccles MP. What is an adequate sample size? Operationalising data saturation for theory-based interview studies. Psychol Heal. (2010) 25:1229–45. 10.1080/08870440903194015 20204937

[102] Barber JA Thompson SG. Analysis of cost data in randomized trials: an application of the non-parametric bootstrap. Stat Med. (2000) 19:3219–36. 10.1002/1097-0258(20001215)19:23&lt;3219::AID-SIM623&gt;3.0.CO;2-P 11113956

[103] Ren S Lai H Tong W Aminzadeh M Hou X Lai S. Nonparametric bootstrapping for hierarchical data. J Appl Stat. (2010) 37:1487–98. 10.1080/02664760903046102

[104] Glick HA Doshi JA Sonnad SS Polsky D. Economic Evaluation in Clinical Trials. Oxford: Oxford University Press (2007).

[105] Van Hout BA Al MJ Gordon GS Rutten FFH. Costs, effects and C/E-ratios alongside a clinical trial. Health Econ. (1994) 3:309–19. 10.1002/hec.4730030505 7827647

[106] Mayer A Zimmermann J Hoyer J Salzer S Wiltink J Leibing E. Interindividual differences in treatment effects based on structural equation models with latent variables: an EffectLiteR tutorial. Struct Equ Model. (2020) 27:798–816. 10.1080/10705511.2019.1671196

[107] Holm S. A simple sequentially rejective multiple test procedure a simple sequentially rejective multiple test procedure. Scand J Stat. (1979) 6:65–70.

[108] Saeb S Zhang M Karr CJ Schueller SM Corden ME Kording KP. Mobile phone sensor correlates of depressive symptom severity in daily-life behavior: AN exploratory study. J Med Internet Res. (2015) 17:e175. 10.2196/jmir.4273 26180009 PMC4526997

[109] Enders CK. Applied Missing Data Analysis. Methodology in the Social. New York, NY: Guilford Press (2010) 377. p.

[110] Birnbaum ML Kulkarni P “Param” Van Meter A Chen V Rizvi AF Arenare E. Utilizing machine learning on internet search activity to support the diagnostic process and relapse detection in young individuals with early psychosis: feasibility study. JMIR Ment Heal. (2020) 7:e19348. 10.2196/19348 32870161 PMC7492982

[111] Chen T Guestrin C. XGBoost: a scalable tree boosting system. In: Proceedings of the ACM SIGKDD International Conference on Knowledge Discovery and Data Mining. New York, NY: Association for Computing Machinery (2016). p. 785–94. 10.1145/2939672.2939785

[112] Sano A Taylor S McHill AW Phillips AJK Barger LK Klerman E. Identifying objective physiological markers and modifiable behaviors for self-reported stress and mental health status using wearable sensors and mobile phones: Observational study. J Med Internet Res. (2018) 20:e210. 10.2196/jmir.9410 29884610 PMC6015266

[113] Sultana M Al-Jefri M Lee J. Using machine learning and smartphone and smartwatch data to detect emotional states and transitions: exploratory study. JMIR mHealth uHealth. (2020) 8:e17818. 10.2196/17818 32990638 PMC7584158

[114] Wshah S Skalka C Price M. Predicting posttraumatic stress disorder risk: a machine learning approach. JMIR Ment Heal. (2019) 6:e13946. 10.2196/13946 31333201 PMC6681635

[115] Domhardt M Steubl L Boettcher J Buntrock C Karyotaki E Ebert DD. Mediators and mechanisms of change in internet- and mobile-based interventions for depression: a systematic review. Clin Psychol Rev. (2020) 83:101953. 10.1016/j.cpr.2020.101953 33422841

[116] Cuijpers P Ebert DD Acarturk C Andersson G Cristea IA. Personalized psychotherapy for adult depression: a meta-analytic review. Behav Ther. (2016) 47:966–80. 10.1016/j.beth.2016.04.007 27993344

[117] Rubel JA Zilcha-Mano S Giesemann J Prinz J Lutz W. Predicting personalized process-outcome associations in psychotherapy using machine learning approaches—A demonstration. Psychother Res. (2020) 30:300–9. 10.1080/10503307.2019.1597994 30913982

[118] Zilcha-Mano S. Toward personalized psychotherapy: the importance of the trait-like/state-like distinction for understanding therapeutic change. Am Psychol. (2020) 10.1037/amp0000629 32658495

[119] Cameron SK Rodgers J Dagnan D. The relationship between the therapeutic alliance and clinical outcomes in cognitive behaviour therapy for adults with depression: a meta-analytic review. Clin Psychol Psychother. (2018) 25:446–56. 10.1002/cpp.2180 29484770

[120] Flückiger C Del Re AC Wampold BE Symonds D Horvath AO. How central is the alliance in psychotherapy? A multilevel longitudinal meta-analysis. J Couns Psychol. (2012) 59:10–7. 10.1037/a0025749 21988681

